# Screening for PPAR Non-Agonist Ligands Followed by Characterization of a Hit, AM-879, with Additional No-Adipogenic and cdk5-Mediated Phosphorylation Inhibition Properties

**DOI:** 10.3389/fendo.2018.00011

**Published:** 2018-02-01

**Authors:** Helder Veras Ribeiro Filho, Natália Bernardi Videira, Aline Villanova Bridi, Thais Helena Tittanegro, Fernanda Aparecida Helena Batista, José Geraldo de Carvalho Pereira, Paulo Sérgio Lopes de Oliveira, Marcio Chaim Bajgelman, Albane Le Maire, Ana Carolina Migliorini Figueira

**Affiliations:** ^1^Brazilian Biosciences National Laboratory (LNBio), Brazilian Center for Research in Energy and Materials (CNPEM), Campinas, Brazil; ^2^Post Graduation Program in Biosciences and Technology of Bioactive Products, Institute of Biology, University of Campinas (UNICAMP), Campinas, Brazil; ^3^Centre de Biochimie Structurale CNRS, Université de Montpellier, Montpellier, France

**Keywords:** peroxisome proliferator activator receptor γ, diabetes, adipogenesis, ligand screening pipeline, non-agonist

## Abstract

Peroxisome proliferator-activated receptor gamma (PPARγ) is a member of a nuclear receptor superfamily and acts as a ligand-dependent transcription factor, playing key roles in maintenance of adipose tissue and in regulation of glucose and lipid homeostasis. This receptor is the target of thiazolidinediones, a class of antidiabetic drugs, which improve insulin sensitization and regulate glycemia in type 2 diabetes. Despite the beneficial effects of drugs, such as rosiglitazone and pioglitazone, their use is associated with several side effects, including weight gain, heart failure, and liver disease, since these drugs induce full activation of the receptor. By contrast, a promising activation-independent mechanism that involves the inhibition of cyclin-dependent kinase 5 (CDK5)-mediated PPARγ phosphorylation has been related to the insulin-sensitizing effects induced by these drugs. Thus, we aimed to identify novel PPARγ ligands that do not possess agonist properties by conducting a mini-trial with 80 compounds using the sequential steps of thermal shift assay, 8-anilino-1-naphthalenesulfonic acid fluorescence quenching, and a cell-based transactivation assay. We identified two non-agonist PPARγ ligands, AM-879 and P11, and one partial-agonist, R32. Using fluorescence anisotropy, we show that AM-879 does not dissociate the NCOR corepressor *in vitro*, and it has only a small effect on TRAP coactivator recruitment. In cells, AM-879 could not induce adipocyte differentiation or positively regulate the expression of genes associated with adipogenesis. In addition, AM-879 inhibited CDK5-mediated phosphorylation of PPARγ *in vitro*. Taken together, these findings supported an interaction between AM-879 and PPARγ; this interaction was identified by the analysis of the crystal structure of the PPARγ:AM-879 complex and evidenced by AM-879’s mechanism of action as a putative PPARγ non-agonist with antidiabetic properties. Moreover, we present an optimized assay pipeline capable of detecting ligands that physically bind to PPARγ but do not cause its activation as a new strategy to identify ligands for this nuclear receptor.

## Introduction

Obesity and type 2 diabetes are characteristic pathologies of the metabolic syndrome and have reached epidemic proportions in recent years (1). They are associated with an increased risk of cardiovascular disease and stroke. Obesity and type 2 diabetes may be defined as accumulation of excessive body fat that impairs health and longevity and as a chronic metabolic disorder that results partly in the inability of the body to respond adequately to circulating insulin, respectively (2). Recent studies have improved our knowledge about these conditions and have suggested that adipose tissue is at the center of the metabolic syndrome (3). Other reports have shown that peroxisome proliferator-activated receptor gamma (PPARγ) plays key roles in adipose tissue, acting as a master regulator of fat cell biology (4–6). Perhaps, the most clinically relevant finding in this field was the now well-established link between PPARγ activity and insulin sensitivity (7).

Peroxisome proliferator-activated receptor gamma is a member of a nuclear receptor superfamily and acts as a ligand-dependent transcription factor. Endogenously, PPARγ is modulated by various fatty acid (FAs) and their metabolites, playing roles in glucose and lipid homeostasis (6, 8–10). To exert its biological effects, PPARγ depends on ligand binding, which induces conformational changes in the receptor’s structure that lead to a dynamic process of corepressor dissociation and coactivator recruitment (11). Through this process, PPARγ activates the transcriptional machinery and regulates the expression of genes involved in several metabolic processes, such as adipocyte differentiation (12, 13) and insulin sensitivity (14, 15).

In this context, PPARγ is the target of thiazolidinediones (TZD), a class of antidiabetic drugs, that improve insulin sensitization and regulate glycemia in type 2 diabetes (16–18). However, despite their beneficial antidiabetic effects, TZD drugs, such as rosiglitazone (Rosi) and pioglitazone, have been associated with several side effects, including weight gain, heart failure, and liver disease, which have restricted the use of these drugs (19–22). In fact, PPARγ activation by TZDs results in dysregulation of the expression of several genes involved in metabolic processes, such as adipogenesis; this dysregulation leads to the undesired side effects. In 2010, Choi and coworkers showed that the insulin-sensitizing effects produced by TZDs were a consequence of the inhibition of cyclin-dependent kinase 5 (CDK5)-mediated PPARγ phosphorylation at serine 273 (or S245 in PPARγ1; the phosphorylation site in PPARγ2 differs from that in PPARγ1 by an additional 28-residue sequence in the N-terminal), rather than the effect of PPARγ activation by these drugs. Accordingly, significant research effort has been made to find selective ligands that modulate PPARγ, promoting its minimum activation and maintaining its positive antidiabetic properties. In this context, the development of PPARγ partial- and non-agonists is a promising strategy for diabetes management.

Hitherto, several screening pipelines have been designed to search for novel PPARγ ligands (23–25). Transactivation assays and combined approaches using transactivation and binding assays are the best-established and used methods to screen for compounds that activate PPARγ (26, 27). However, only a restricted number of screening techniques have been designed and applied to find PPARγ non-agonists (28, 29). Thus, in the present study, we developed a screening pipeline to identify ligands capable of interacting with PPARγ but that lack the activation properties ascribed to its full agonists; the pipeline developed was based on previously reported approaches (29, 30). Moreover, we characterized a previously identified compound, AM-879 (29), as a putative PPARγ non-agonist, describing in detail its mechanism of action. AM-879 did not exhibit adipogenic properties and could inhibit CDK5-mediated phosphorylation of PPARγ.

## Materials and Methods

### Screening Compounds Selection

A library of 80 compounds composed of different classes of molecules, including sulfonamides, sulfonylureas, hydrazones, chalcones, and TZD, was used for PPARγ ligand screening. This mini-library was composed by 67 compounds, which previously had presented some antidiabetic properties in mice (personal communication), and that were synthesized and generously gifted by Prof. Dr. Ricardo José Nunes and Prof. Dr. Rosendo Augusto Yunes from UFSC (Brazil); one compound gifted by the Laboratory of Chemistry and Natural Products (LQPN, LNbio/CNPEM, Brazil); six gifted by Prof. Dr. Ronaldo Aloise Pilli from UNICAMP (Brazil), and six commercial compounds, which includes the already described PPARγ ligand, AM-879 (4-({2-[(1,3-dioxo-1,3-dihydro-2H-inden-2-ylidene) methyl] phenoxy} methyl) benzoic acid, AM-879-40965082, Specs).

### PPARγ Expression, Purification, and Biophysical Characterization

Peroxisome proliferator-activated receptor gamma ligand-binding domain (LBD) construction (isoform PPARγ1), encoding AAs 207–477, inserted in pET 28a (+), was expressed in *Escherichia coli* strain BL21 (DE3). Cells were growth in LB, at 22°C, until OD_600nm_ reached 0.8, and were induced with 1 mM Isopropyl β-d-1-thiogalactopyranoside (IPTG) for 16 h. After this, bacteria were harvested by centrifugation (16,000 rcf), and the pellet was resuspended in lysis buffer (50 mM Tris–HCl pH 8.0, 300 mM NaCl, 5% glycerol, 2 mM β-mercaptoethanol, 0.1 mM PMSF, 1 mg lysozyme). The extract was sonicated on ice bath and the soluble fraction was separated by centrifugation at 36,000 rcf, for 40 min, at 4°C, in Avanti J26xPT (Beckman Coulter, rotor JA-25-50). Purification was performed by affinity chromatography using a Talon Superflow resin (Clontech), the protein was eluted in 50 mM Tris–HCl pH 8.0, 300 mM NaCl, 5% glycerol, 300 mM imidazole and 2 mM β-mercaptoethanol, and submitted to a size exclusion chromatography through a Hiload Superdex 200 16/60 (GE life sciences), following the manufacturer’s instruction, to improve purity and remove imidazole. Samples purity was checked by SDS-PAGE and protein concentration was measured through nanodrop (ThermoScientific). For crystallization, purified PPARγ LBD (construction 197-477) was concentrated to 8.5 mg/ml in a buffer containing 20 mM Tris–HCl, pH 8.5, 250 mM NaCl, 5 mM dithiothreitol, and 1 mM EDTA.

Sample aggregation, monodispersity and oligomeric state were evaluated after protein purification by performing dynamic light scattering (DLS), native electrophoresis (NG), and circular dichroism (CD) measurements (Figure S1 in Supplementary Material). DLS measurements were obtained in Protein Solutions DynaPro DLS system (Wyatt), with 50 accumulations of 2.5 s, at 10°C. The NG was performed in phast system (GE Life Sciences), using 8–25% acrylamide gradient phast gels, following the manufacturer’s manual. Far-UV CD measurements were performed on a Jasco J-810 Spectropolarimeter coupling to a peltier control, in 1 mm Quartz cuvettes (Hellma) and protein samples (5 μM) were dialyzed to remove salt excess in Slide-A-Lyzer^®^ Mini Dialysis Units tubes 10,000 MWCO, for 16 h. The spectra were recorded from 190 to 260 nm, at 10°C, using a 50 nm/min scan speed and 20 accumulations.

### Thermal Shift Assay (TSA)

Protein thermal unfolding was monitored by measuring the SYPRO^®^ Orange probe (Life technologies) fluorescence. PPARγ samples (4 μM) were incubated with threefold ligand excess (12 μM) and SYPRO Orange, in a reaction volume of 25 μL. Rosi was used as a PPARγ full agonist control and, dimethyl sulfoxide (DMSO), as apo-PPARγ control. TSA was performed in triplicates, in 96-well PCR microplates (Applied Biosystems—Life Technologies), through an RT-PCR device (7500 Real Time PCR System). Samples were heated at 1°C per min, from 10 to 90°C. Fluorescence intensities were plotted as a function of temperature and, the melting temperature (Tm) of the protein unfolding transition was obtained through a Boltzmann model in Origin Pro 8.1 and using an Excel based worksheet (DSF Analysis v3.0.2, ftp://ftp.sgc.ox.ac.uk/pub/biophysics).

### NanoDSF

Intrinsic tryptophan and tyrosine fluorescence were measured using NT48 device (NanoTemper), as a more sensitive technology to check protein stability in comparison to TSA. Capillaries were filled with 10 μL of PPARγ (25 μM), pre-incubated with DMSO or ligands (75 μM, 3 M excess) for 1 h, on ice. The initial fluorescence scan of loaded capillaries was performed at 20°C, monitoring emission wavelength at 330 and 350 nm in order to find the optimal signal range. After this step, samples were subjected to a temperature ramp of 1°C/min from 20 to 90°C and fluorescence was constantly monitored. Data were analyzed and Tm was determined using PR. ThermControl software (NanoTemper).

### 8-Anilino-1-Naphthalenesulfonic Acid (ANS) Fluorescence Quenching

8-Anilino-1-naphthalenesulfonic acid fluorescence quenching assay was performed incubating 1 μM of PPARγ with 10 μM of the fluorescent probe ANS for 30 min. Following this, increasing concentrations (0.1–60 μM) of the pre-selected 20 compounds were titrated in protein-ANS solution. The fluorescence measurements were taken on a EnSpire multimode plate reader (Perkin Elmer) through a 96-well all-black-walled plate (Perkin Elmer), with excitation wavelength set at 380 nm and emission wavelength range of 400–600 nm, at 25°C. Fluorescence emission at the wavelength of maximum intensity (475 nm) was monitored for each concentration of compounds. Fluorescence data were fitted to binding curves using Hill model for dissociation constant (Kd) calculation. All experiments were performed in triplicates and data were processed using the software Origin Pro 8.1.

### Cell Transactivation Assay

293T (ATCC^®^ CRL-3216™) cells were cultured in Dulbecco’s Modified Eagle’s Medium (DMEM, Gibco^®^) supplemented with 10% (v/v) fetal bovine serum, 50 U/mL penicillin, and 50 mg/mL streptomycin, at 37°C, and humidified atmosphere of 5% CO_2_. For transactivation assays, cells were seeded in 24-well plates (density of 2.0 × 10^5^ cells/well), and transiently transfected with Lipofectamine^®^ 2000 (Invitrogen) and 0.5 μg of the following plasmids: pRL-TK, a transfection control, which express *Renilla reniformis* luciferase constitutively; pGRE-LUC, which contains responsive element for GAL4 protein followed by reporter gene of firefly luciferase; pBIND-PPARγ, a chimeric protein composed by GAL4-DBD and PPARγ-LBD, under control of the cytomegalovirus promoter (CMV), in a proportion of 1.5 μg DNA:2 μL of Lipofectamine. As negative control, empty BlueScript plasmid was used instead of pBIND-PPARγ. For isotype specificity transactivation assay, cells were seeded in 96-well plates (density of 4.0 × 10^4^ cells/well) and transfected with pRL-TK; pGRE-LUC and pBIND-PPARα; and pBIND-PPARβ.

Four hours after transfection, the culture medium was exchanged to DMEM plus 10% FBS Charcoal stripped broth and 1 μM of each tested compound, Rosi or 1% DMSO was added to each well. In dose–response experiments, concentration of compounds was increased from 0.01 to 0.1 mM. After 24 h of treatment, reporter gene expression was measured with Dual-Luciferase^®^ Reporter (DLR™) Assay System (Promega), according to manufacturer’s instructions in a GloMax-Multi + Detection System (Promega). Average curves were fitted applying Boltzmann function until fitting converges with a software tolerance criterion using Origin software (version 8.0, OriginLab Corporation).

### *In Vitro* Coregulators Recruitment

Apo-PPARγ or PPARγ in the presence of 3 M excess of Rosi (positive control), P11 or AM-879, in concentration that varies from 0.004 to 20 μM was incubated with 20 nM of fluorescein labeled TRAP peptide (ID2) or NCoR peptide (ID2). These mixtures were submitted to fluorescence anisotropy measurements using ClarioStar^®^ plate reader (BMG), with emission and excitation filters adjusted for fluorescein. Kd were obtained from fluorescence data fitted to binding curves using Hill model (Figure S4 in Supplementary Material). Data from experiment done in triplicate were compared by the one-way ANOVA followed by Bonferroni’s multiple comparison test using GraphPad Prism.

### Adipocyte Differentiation Assay

3T3-L1 (ATCC^®^ CRL-3242™) cells were cultured in DMEM (Gibco^®^) supplemented with 10% (v/v) fetal bovine serum, 50 U/mL penicillin, and 50 mg/mL streptomycin, at 37°C, and humidified atmosphere of 5% CO_2_. After achieved 70% confluency, cells were trypsinized and seeded in 6-well plates (Corning) at a density of 2.8 × 10^5^ cells/well. After 2 days, cells were treated with dexamethasone (1 mM) to induce preadipocytes differentiation, and 1 μM of Rosi, 1 μM of AM-879, or 1% DMSO. After treatment, the culture medium was exchanged every 48 h, supplemented with 1 μM of each ligand, for 6 days. In order to measure adipocyte differentiation, fully differentiated 3T3-L1 cells were washed with phosphate-buffered saline (PBS), fixed with 10% formalin (v/v) for 1 h, and then, stained with oil red solution (Oil Red O) (0.3% in isopropanol 60%) for 20 min. After staining, cells were washed with PBS and photographed in Nikon MTS microscope (Nikon). Lipid accumulation was also quantified by Oil Red O absorbance measurement at 520 nm. For this measurement, cells lysis was performed in 200 μL of isopropanol and 4% Igepal solution, and the absorbance of the samples was recorded in a 10-mm cuvette in a spectrophotometer (V-530, Jasco). Data were compared using unpaired *t*-test.

### Gene Expression Analysis

Total RNA from differentiated 3T3-L1 cells was extracted by RNeasy kit (Qiagen) and cDNAs were synthesized with High-Capacity cDNA Reverse Transcription Kit (ThermoFischer Scientific). Quantitative PCR reactions were performed with SYBR Real Time PCR master mixes (ThermoFischer Scientific) and 50 ng of cDNA, in a 7500 Real Time PCR system (Applied Biosystems). Relative mRNA expression was determined by the ΔΔ-Ct method normalized to GAPDH levels. The sequences of primers used in this study are presented in Table S2 in Supplementary Material.

### *In Vitro* Phosphorylation Assay

Peroxisome proliferator-activated receptor gamma phosphorylation mediated by CDK5 at serine 273 was measured by luminescent detection of ADP produced in a *in vitro* phosphorylation reaction. Purified PPARγ LBD (1.2 μM) was previously pre-incubated with 5 M excess of Rosi, AM-879 or DMSO, for 30 min, at 4°C. Then, protein and ligands were incubated with 50 ng CDK5/p35 (Sigma), for 1 h, at room temperature, in the CDK5/p35 reaction buffer (composition recommended by manufacturer) and in the presence of ATP 10 μM. After reaction, ADP detection was performed by using ADP-Glo™ kinase assay (Promega) following manufacturer’s instructions. Luminesce signal was recorded using GloMax-Multi + Detection System (Promega) microplate luminometer.

### Crystallization, Crystallographic Data Collection, Processing, and Structure Refinement

Crystallization was performed as described in Ref. (31). Crystals were obtained by co-crystallization, mixing 1 μL protein solution (8.5 mg/mL PPARγ LBD), 1 μL well solution (0.9 M trisodium citrate, 100 mM HEPES, pH 7.0, 3.5% 1,2-propanediol) and AM-879 in a molar ratio of 1:4 (protein:ligand) in the drop. After 2–3 days crystals appeared and, within a few days, grew to 150–200 μm. Before diffraction data collection, crystals were transferred to a cryoprotectant containing 20% glycerol in well solution, supplemented with fourfold excess ligand, and frozen in liquid nitrogen. Diffraction data were collected at the MX2 beamline from the Laboratorio Nacional de Luz Sincrotron (LNLS, Campinas, Brazil). Diffraction data were processed using XDS (32). Structures were solved by using the previously reported structure 3PBA (33) from which the ligand was omitted. The calculation of POLDER map (34) was necessary to position the ligand into the electron density. The structure was modeled with COOT (35) and refined in phenix.refine from the PHENIX program suite (36). The atomic coordinates have been deposited in the Protein Data Bank under the accession code 6AN1.

## Results

### Structure Stabilization As a First Tool for PPARγ Ligand Search

**Figure 1:**
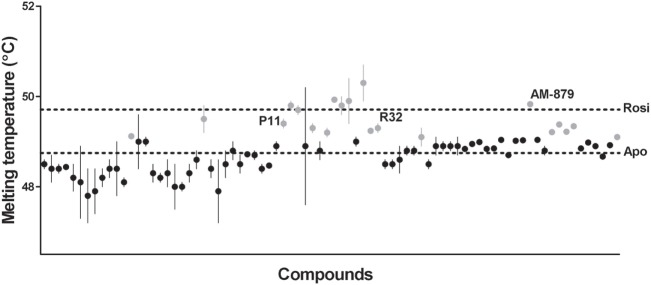
Melting temperature of peroxisome proliferator-activated receptor gamma (PPARγ) obtained by thermal shift assay (TSA) in the presence of 80 screened compounds. Upper dashed line marks the melting temperature (Tm) of PPARγ in presence of Rosiglitazone (Rosi; positive control) and the lower dashed line marks the Tm of apo-PPARγ (negative control). Gray circles represent the 20 selected compounds with highest Tm, and the black circles correspond to the unselected compounds. Tm of the three ligands selected at the end of screening are indicated by their names P11, R32, and AM-879. thermal shift assay was performed in triplicates in three independent experiments, errors bars represent SEM.

Ligand-bound PPARγ presents a higher structural stability than the unbound receptor (37, 38); therefore, the first step in our ligand screening involved searching for molecules that increased the stability of PPARγ using a TSA. Tms obtained for PPARγ in the presence of each compound are shown in Figure 1. Apo-PPARγ presented a Tm of 48.75 ± 0.08°C (lower line), while in the presence of Rosi, the Tm shifted to 49.71 ± 0.05°C (upper line). Among all the 80 tested compounds, we selected 20 that presented the most significant increases in Tm (gray circles in Figure 1). These molecules promoted the receptor tertiary structure stabilization, presumably by binding to PPARγ; therefore, they were chosen for further investigation in our proposed pipeline.

### Measurements of Direct Binding to Three Possible Selected Ligands for PPARγ

A PPARγ ligand-binding assay was employed as the second step of the pipeline; this assay was based on the fluorescence quenching of ANS. The ANS probe interacts with the PPARγ binding pocket (30); therefore, the fluorescence quenching observed after titration of a compound is a consequence of the successful competition the compound and the ANS probe in the receptor ligand-binding pocket (LBP), suggesting that the compound effectively binds to PPARγ.

**Figure 2:**
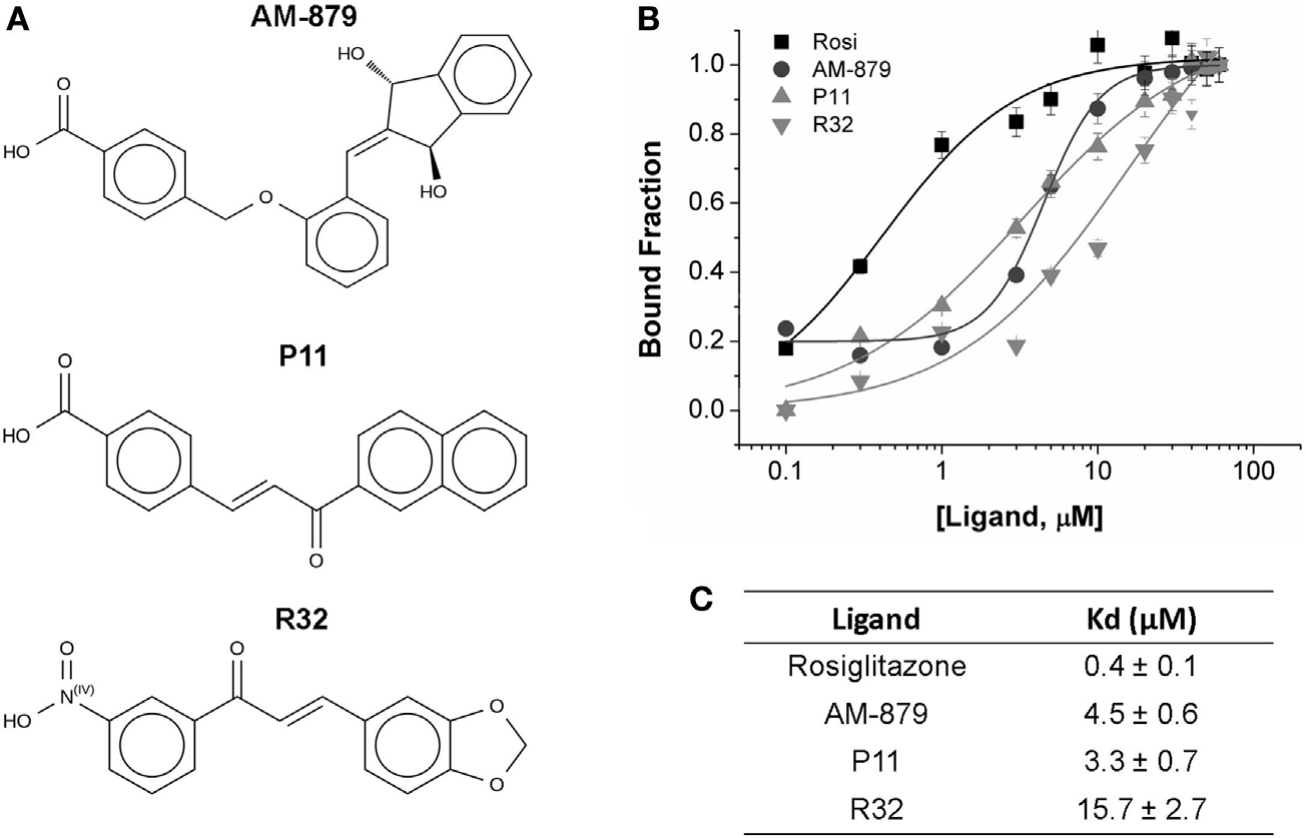
Binding affinity of AM-879, P11, and R32 ligands to peroxisome proliferator-activated receptor gamma (PPARγ) ligand-binding domain (LBD). **(A)** Molecular structure of the selected ligands. **(B)** PPARγ LBD bound to increased concentrations of Rosiglitazone (Rosi), AM-879, P11, and R32 (until 60 μM). Bound fractions were determined from 8-Anilino-1-naphthalenesulfonic acid (ANS) fluorescence signal decayment at 340 nm, as a result of ANS probe displacement from PPARγ pocket, and normalized from 0 to 1. Data are the mean ± SEM (*n* = 3). **(C)** Dissociation constants (Kd, in μM) calculated from ligand-binding curves fitted through Hill model using Origin.

From the 20 compounds pre-selected by the TSA, we identified three that significantly decreased the ANS fluorescence intensity in a concentration-dependent manner; this concentration-dependent decrease is also observed on treatment with Rosi (Figure S2 in Supplementary Material). Compound AM-879, and two chalcones, P11 and R32 (Figure 2A), showed total suppression of the fluorescence signal and a reasonable affinity for the receptor binding site (Figure 2C).

Analysis of the fluorescence intensities at 475 nm vs the ligand concentration confirmed that Rosi had the highest affinity for PPARγ, with a Kd of 0.4 ± 0.1 μM, while AM-879 and P11 showed moderate affinities (Kd values of 4.5 ± 0.6 and 3.3 ± 0.7 μM, respectively). Among the selected compounds, R32 exhibited the lowest affinity for PPARγ (Kd of 15.7 ± 2.7 μM) (Figures 2B,C).

In addition, we employed a nano differential scanning fluorimetry (nanoDSF) assay to confirm the stability of the structure when these three ligands bind PPARγ (Table S1 in Supplementary Material). The Tms obtained by nanoDSF maintained the same order for the tested ligands as that obtained by TSA. A higher Tm (49.9 ± 0.027°C) was induced by Rosi, followed by the ligands AM-879 (48.8 ± 0.114°C) and P11 (48.0 ± 0.172°C). The difference between the PPARγ Tm in the presence of DMSO and in the presence of Rosi was 2.6°C. Thus, we confirmed the binding of AM-879, P11, and R32 to PPARγ ligand-binding domain (LBD) in the micromolar range; therefore, we further characterized the effects of these molecules on PPARγ activation.

### Transactivation Assay Shows No PPARγ Activation by AM-879 and P11 but a Slight Activation by R32

**Figure 3:**
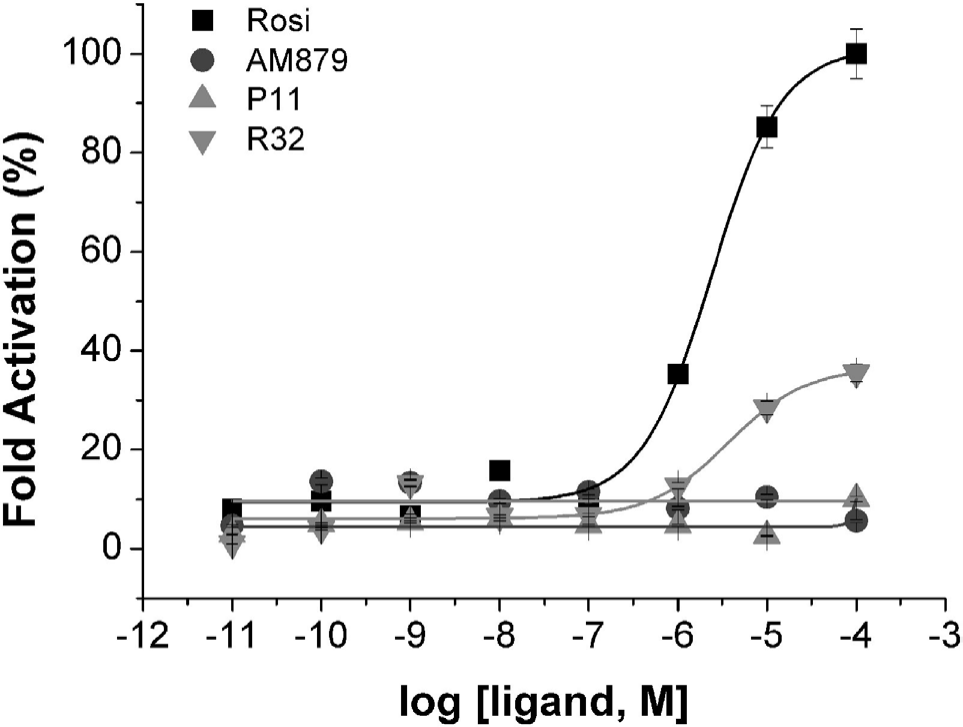
Peroxisome proliferator-activated receptor gamma (PPARγ) transactivation assay in the presence of the three selected ligands in 293T cells using a GAL4-PPARγ-LBD reporter gene. Concentration-effect curves were measured for AM-879 (circles), P11 (triangles), and R32 (inverted triangles) ligands. Rosiglitazone (Rosi, squares) was used as positive control. Ligand concentration varied from 0.01 nM to 0.1 mM and the response was presented as fold activation, normalized against the maximal luciferase induction produced by Rosi. Data are the mean ± SEM (*n* = 3).

To identify compounds that bind to PPARγ and bring about minimal activation of this receptor, we performed a transactivation assay in 293T cells as the last step of our pipeline. ANS and TSA assays showed that AM-879, P11, and R32 have lower affinity for PPARγ than Rosi; therefore, we verified whether increasing concentrations of these compounds could induce activation of PPARγ in a concentration-dependent manner. The dose–response curves shown in Figure 3 indicated that Rosi activated PPARγ the most, with an EC_50_ of 2.39 ± 0.01 μM, while AM-879 and P11 did not activate PPARγ even at the highest concentration. By contrast, compound R32 showed slight activation at concentrations above 1 μM, with an EC_50_ of 3.65 ± 0.03 μM, which is 35% of the maximal activation mediated by Rosi. In consequence, we classified this ligand as a partial agonist and did not include it on our further biological and structural characterizations. In addition, we also investigated the isotype selectivity of AM-879, P11, and R32. Interestingly, none of them activated other PPAR isotypes (PPARα and PPARβ), in comparison to their full agonists (Figure S3 in Supplementary Material). In conclusion, R32 seems to be a partial agonist because it slightly but significantly activates PPARγ, whereas AM-879 and P11 are not able to activate any PPARs in 293T cells. For this reason, we decided to include only AM-879 and P11 in further biological and structural characterization studies.

### AM-879 Differs from P11 in Its Ability to Recruit a Corepressor

We next investigated the influence of AM-879 and P11 on the recruitment of coregulators by PPARγ. For this, we performed *in vitro* binding assays using fluorescence anisotropy to measure PPARγ affinities for coactivator (TRAP SRC2) or corepressor (NCOR ID2) peptides in the presence of an excess of these two ligands, with Rosi as the control.

**Figure 4:**
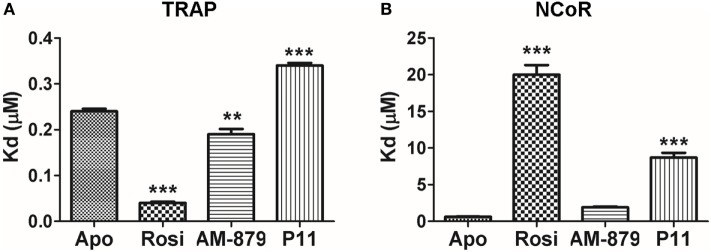
*In vitro* recruitment of coregulators by peroxisome proliferator-activated receptor gamma (PPARγ) in the presence of AM-879 and P11. **(A)** Dissociation constants (Kds, μM) between PPARγ ligand-binding domain (LBD) and TRAP coactivator peptide in the absence (Apo) or in the presence of the ligands Rosiglitazone (Rosi), AM-879 and P11. **(B)** Kd (μM) between PPARγ LBD and NCoR corepressor peptide in the absence (Apo) or in the presence of ligands. Kds were determined from ligand-binding curves (until 20 μM), obtained by fluorescence anisotropy measurements using fluorescein labeled TRAP (ID2) or NCoR (ID2) peptides (20 nM). Data are the mean ± SEM (*n* = 3, **p < 0.05, ***p < 0.001 vs Apo).

As expected, the full agonist Rosi efficiently increased the affinity of PPARγ for the TRAP coactivator peptide (Kd of 0.040 ± 0.005 μM) in comparison to the apo form (Kd of 0.24 ± 0.01 μM). In turn, the Kd in the presence of AM-879 and P11 was close to that measured for apo-PPARγ (0.19 ± 0.02 and 0.34 ± 0.05 μM, respectively, Figure 4A), indicating a more similar behavior to the unbound receptor. This experiment showed that AM-879 does not favor coactivator recruitment, whereas P11 only slightly induced coactivator recruitment.

Rosiglitazone strongly decreased the affinity of PPARγ for the NCOR corepressor peptide (Kd of 20.0 ± 2.3 μM) compared with the apo form (Kd of 0.61 ± 0.07 μM) (Figure 4B), as expected for a full agonist. By contrast, the presence of AM-879 did not induce any release of the NCOR peptide; the Kd value measured in this condition was in the same range as that measured for the apo form (Kd of 1.9 ± 0.2 μM) (Figure 4B). In the presence of P11, the interaction between PPARγ and the corepressor peptide lowered to reach a Kd of 8.7 ± 1.1 μM. This suggests that although ligand P11 did not activate the receptor, it is not a good enough ligand to keep PPARγ in the repressed state. Thus, we identified two PPARγ ligands that did not activate the receptor and did not promote coactivator binding. However, P11 failed to maintain PPARγ bound to the corepressor, and, thus, we decided not to further characterize this ligand. Interestingly, despite AM-879 having already been described as PPARγ ligand (29), we found that it is not able to induce coactivator recruitment or corepressor release from PPARγ. For this reason, we chose to further characterize the mechanism of action of AM-879, in terms of adipocyte differentiation, PPARγ Ser273 phosphorylation, and gene expression.

### AM-879 Does Not Induce Adipocyte Differentiation and Induces a Different Profile of Gene Expression in 3T3-L1 Cells Compared with That Induced by Rosi

We next investigated the action of AM-879 on endogenous genes by studying its ability to induce adipogenesis, a well-characterized PPARγ-regulated function (39).

**Figure 5:**
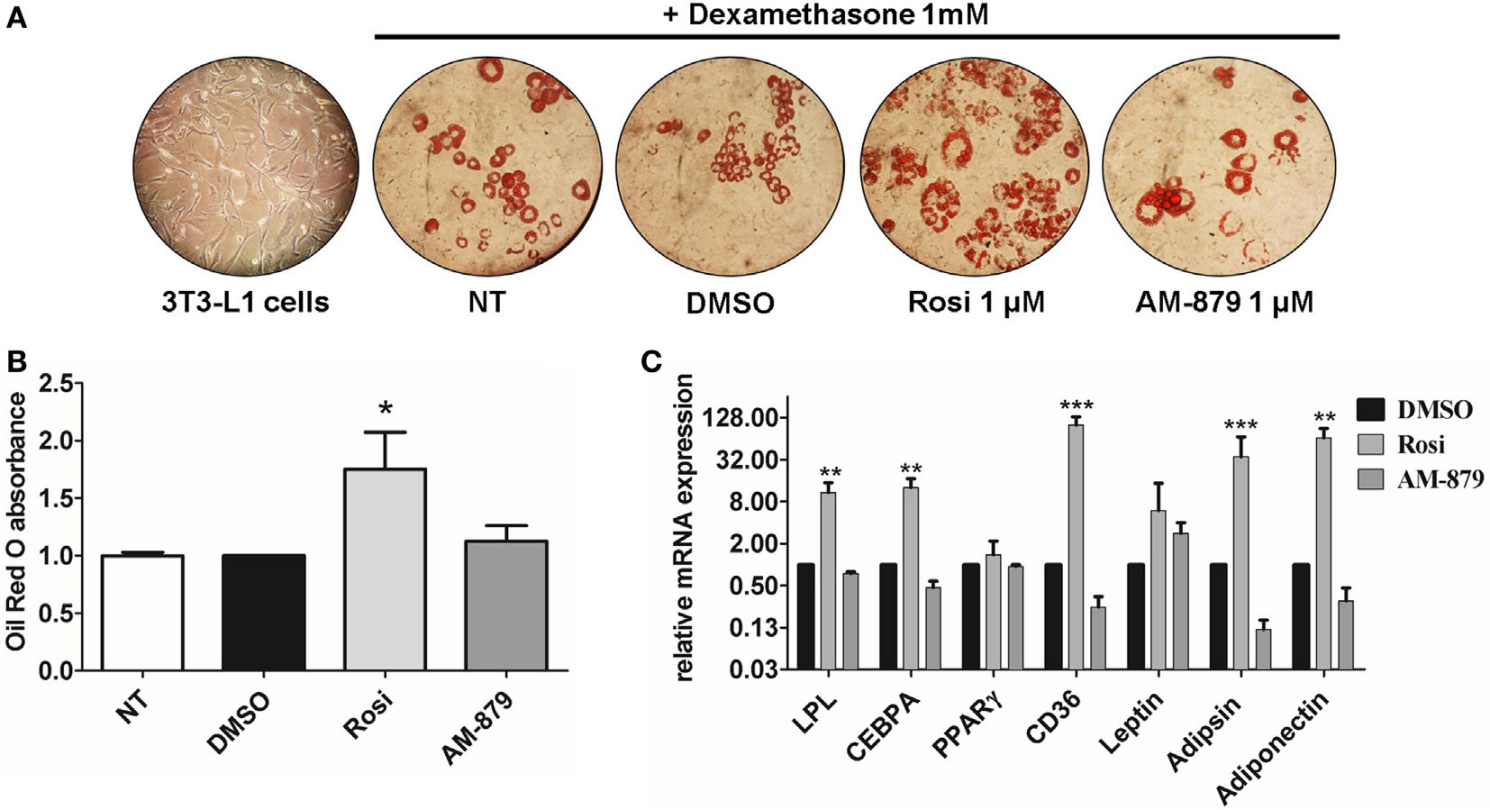
Effects of AM-879 on adipocyte differentiation and gene expression. **(A)** Representative images (400×) of lipid accumulation by Oil Red O staining in 3T3-L1 differentiated cells under different conditions. Images were obtained in random fields of three independent experiments performed in duplicates. **(B)** Quantification of lipid accumulation by Oil Red O absorbance measurement at 520 nm in not-treated 3T3-L1 differentiation cells, or in cells treated with dimethyl sulfoxide (DMSO) (control, 5%), Rosiglitazone (Rosi, 1 μM), or AM-879 (1 μM). Data were normalized by DMSO absorbance. **(C)** RT-qPCR analysis of *LPL, CEBPA, PPAR*γ, *CD36, Leptin, Adipsin*, and *Adiponectin* expression levels in differentiated 3T3-L1 cells treated with 1 μM Rosi, 1 μM AM-879, or 1% DMSO (control). Data are the mean ± SEM (*n* = 3 independent replicates, *p < 0.05, **p < 0.01, ***p < 0.001 vs DMSO).

3T3-L1 preadipocytes treated with dexamethasone were able to differentiate into adipocytes (Figure 5A) and, as expected, the addition of the full PPARγ agonist Rosi strongly induced adipogenesis, as evidenced by oil red O staining (Figures 5A,B). In contrast to Rosi, ligand AM-879 did not induce a significant increase in adipocyte differentiation or lipid accumulation (1.12 ± 0.13-fold) in comparison with DMSO (Figures 5A,B).

Moreover, the effect of AM-879 on adipocyte differentiation was further studied by examining the endogenous expression of a number of PPARγ target genes, namely *LPL* (lipoprotein lipase), *Cebpa, Pparg* (PPARγ) itself, *Cd36, Lep* (Leptin), *Cfd* (Adipsin), and *Adipoq* (Adiponectin), in 3T3-L1 cells. Cells treated with AM-879 presented slightly reduced levels of *Cfd, Adipoq*, and *Cd36* expression, in comparison to the negative control (DMSO), while Rosi strongly increased the relative expression of these genes. By contrast, AM-879 did not alter the relative expression levels of *Pparg, Lep, Cebpa*, and *Lpl* (Figure 5C). These results showed that AM-879 induce a different change in the gene expression profile compared with that induced by Rosi.

### AM-879 Reduced PPARγ Phosphorylation Induced by CDK5

AM-879 did not activate PPARγ; therefore, we investigate if it was capable of interfering with PPARγ phosphorylation at Ser 273. The inhibition of CDK5-mediated phosphorylation of PPARγ is a well-known antidiabetic mechanism produced by drugs, such as Rosi (6).

**Figure 6:**
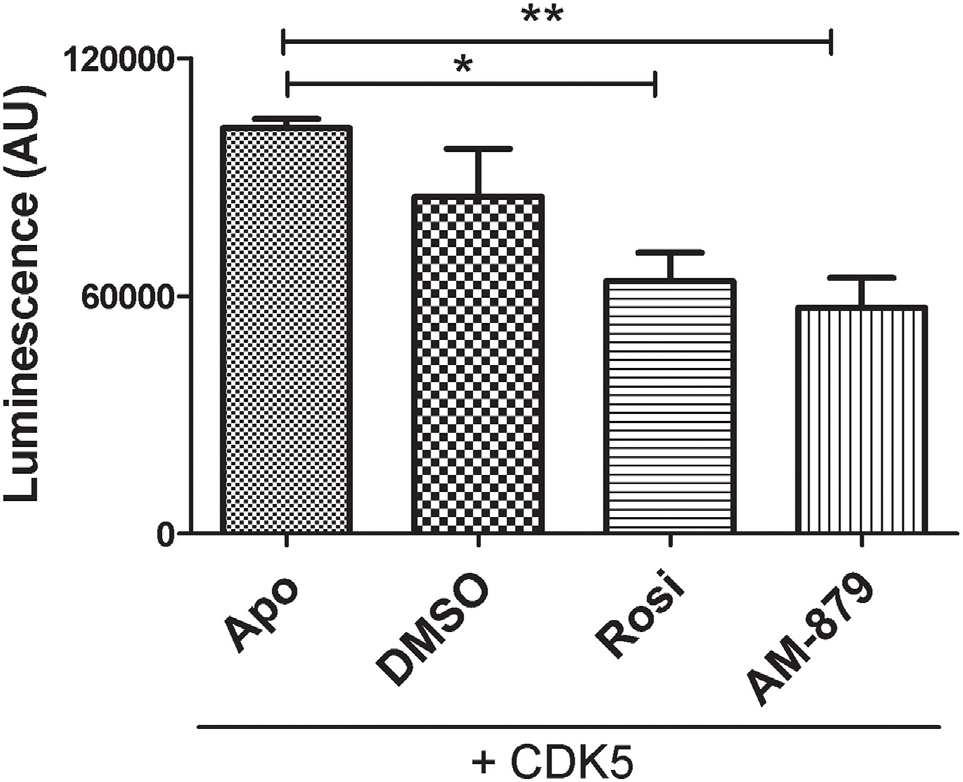
*In vitro* inhibition of cyclin-dependent kinase 5 (CDK5)-mediated phosphorylation of peroxisome proliferator-activated receptor gamma (PPARγ) by AM-879. Luminescence signal detected as consequence of ADP production in *in vitro* phosphorylation reaction containing CDK5/p35 kinase, ATP and PPARγ ligand-binding domain (ADP-Glo™ Kinase assay). Reactions were conducted in the absence (Apo) or the presence of threefold excess of Rosiglitazone (Rosi), AM-879 or dimethyl sulfoxide (DMSO). Data are the mean ± SEM (*n* = 3, *p < 0.05, **p < 0.01, vs Apo).

Using a luminescent ADP detection assay, we detected the ADP produced by CDK5-mediated phosphorylation of PPARγ. As expected, preincubation of Rosi with PPARγ decreased the phosphorylation of this receptor by 22% (Figure 6). Interestingly, ligand AM-879 was more effective (28%) at reducing the phosphorylation of Ser 273 induced by CDK5 compared with Rosi. Thus, in addition to being a non-agonist of PPARγ, ligand AM-879 is a more efficient inhibitor of CDK5-induced PPARγ phosphorylation; therefore, AM-879 may promote insulin sensitization.

### Crystal Structure of the PPARγ:AM-879 Complex Supports Its Non-Agonist Activity of AM-879

**Figure 7:**
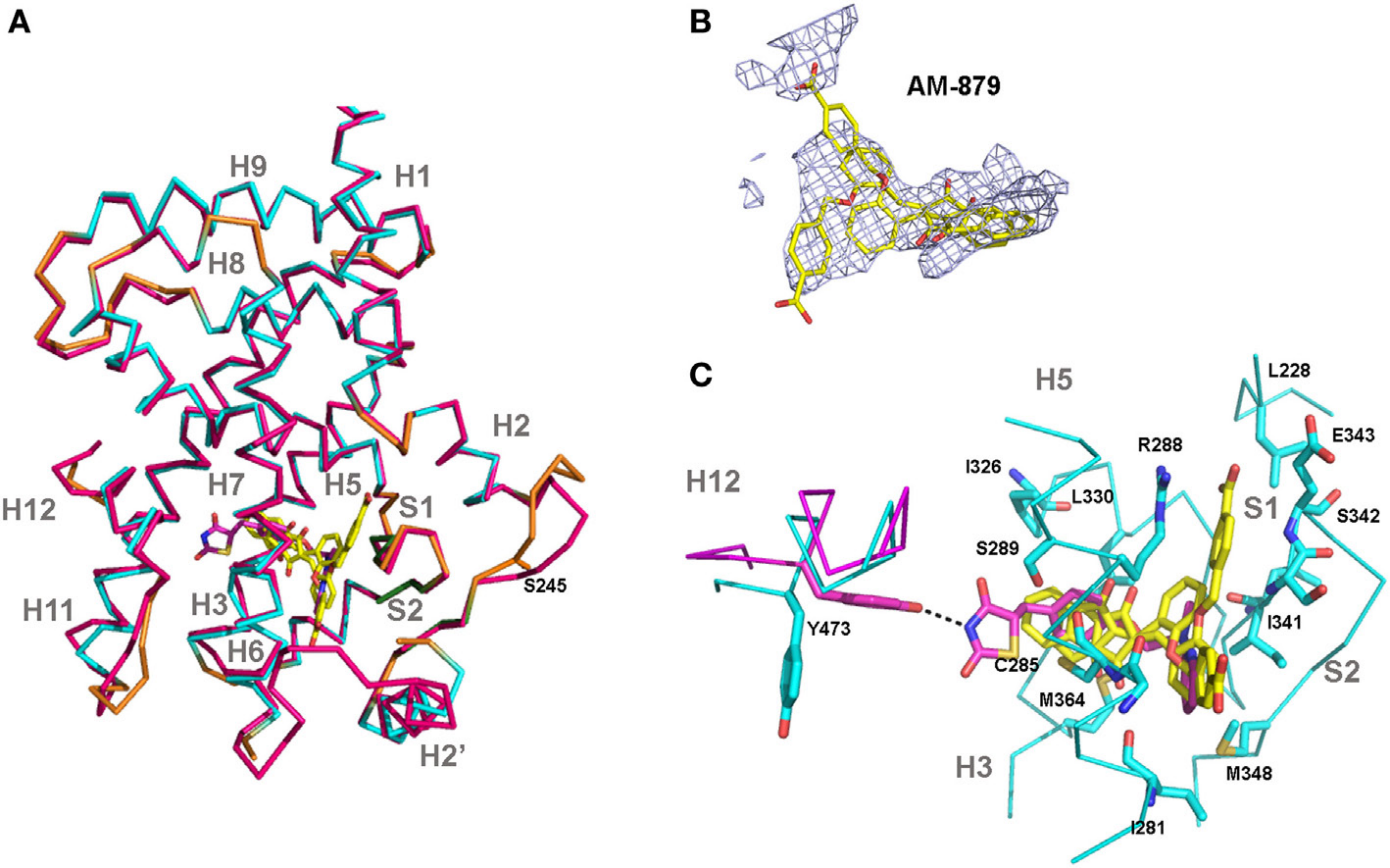
Crystal structure of peroxisome proliferator-activated receptor gamma (PPARγ)-ligand-binding domain (LBD) in complex with AM-879. **(A)** Superimposition of the co-crystal structure of PPARγ-LBD bound to AM-879 on the structure with Rosiglitazone (Rosi; PDB code 2PRG). Carbon atoms are shown in cyan (alpha helix), green (beta-sheet), and orange (loop) in the AM-879 complex, and in pink, in the Rosi structure. The ligands are shown in stick representation with carbon colored yellow or pink, respectively, for AM-879 and Rosi and the serine S273, site of phosphorylation by cyclin-dependent kinase 5 (CDK5), is shown as orange sticks. **(B)** AM-879 in the Polder map contoured at 3.0 σ. **(C)** PPARγ residues (shown as blue sticks) in contact with AM-879. Highlight for Tyrosine 473 (pink sticks) important for the Rosi binding and agonism. Residues number corresponds to PPARγ1 isoform.

Finally, we solved the crystal structure of PPARγ LBD bound to AM-879 to reveal the mechanism by which this compound binds to PPARγ without inducing its activation. The 2.7 íresolution structure of the complex between PPARγ and AM-879 derived from co-crystals reveals the canonical fold of the agonist conformation of nuclear receptor LBDs (40) (Figure 7A; Table S3 in Supplementary Material). In fact, the structure is nearly identical to that of PPARγ in complex with Rosi (PDB code 2PRG) (41), with a root-mean-square deviation value of 0.422 A, calculated for 216 Cα atoms. The most striking changes were in the loop between helices H2 and H3. The electron density corresponding to AM-879 is rather poorly defined, but many trials of co-crystallization with different excesses of ligand and of soaking did not produce a better density for the ligand. However, the calculation of a Polder map (34) and its statistical analysis (0.6772 for the calculated Fobs (observed *F* value) with the ligand compared to 0.3777 for the calculated Fobs without the ligand) supported the view that the ligand is present in the β-sheet sub-pocket of PPARγ and extends between C285 and M364 (Figure 7B). The electron density map presents three regions in which we could fit the ligand in different orientations. One model of the complex could be calculated with two alternative conformations of the ligand (Figure 7B), with reasonable statistically significant differences (R/Rfree = 22.8%/28.7%). However, with these data, we cannot determine whether the models result from truly different ligand conformations or involve the unique placement of the ligand in one conformation.

Based on existing PPARγ complex structures, it has been suggested that full agonists, such as Rosi, occupy both the H12 and β-sheet sub-pockets, establishing hydrogen bonds with residues Y473 (H12), on the one side, and S342 (S1/S2), on the other side, whereas partial agonists or non-agonists would bind essentially to the β-sheet sub-pocket (42). Our ligand AM-879 occupied the β-sheet sub-pocket establishing a contact with S342 and only a small part of the H12 sub-pocket, with no direct interaction with H12; these interactions are in accordance with its non-agonist properties. In addition, the limited number of hydrogen bonds and van der Walls interactions established with PPARγ residues could account for the low affinity of this ligand for the protein (Figure 7C). Thus, the poorly defined density of the ligand suggests a dynamic behavior of this compound, which is not well stabilized in the LBP.

## Discussion

In this study, we developed a new approach to screen for PPARγ ligands following a pipeline of assays that consider the biophysical characteristics of this nuclear receptor, aiming to identify ligands that bind to the receptor but do not promote coactivator recruitment, phosphorylation of S273, and adipogenic activity. Based on this, we first identified three hit compounds (AM-879, P11, and R32) with partial or non-agonist properties on PPARγ. Moreover, we characterize one of them deeply, compound AM-879, as a promising PPARγ non-agonist. Despite the fact that this ligand was previously described (29), our results showed, for the first time, that AM-879 could inhibit CDK5-mediated PPARγ phosphorylation and did not exhibit adipogenic effects; both these observations were supported by the analysis of the PPARγ:AM-879 co-crystal structure.

From an initial library of 80 compounds, our screening selected three of them because of their ability to stabilize the receptor and bind to the PPARγ hydrophobic pocket. Among the 20 compounds that efficiently stabilized the PPARγ structure (Figure 1), 17 compounds, including the one that induced the highest Tm of PPARγ in TSA assay, were not able to significantly displace the ANS probe in the second step of screening (Figure S2 in Supplementary Material). This evidence suggests that, despite TSA being a primary filter for ligand screening, it is strongly recommended to run a secondary assay, whose objective is finding ligands that interact with the LBP of the protein.

In addition, there are questions surrounding the application of TSA as the best screening assay to find PPARγ ligands. First, it was reported (29) that PPARγ TSA produces, in some cases, Tm values similar for both the bound and unbound state of the receptor; this could lead to misinterpretation of data. Furthermore, it was noted that the difference between the Tms of the apo and compound-bound forms of PPARγ could not be estimated correctly due to the presence of free FAs from bacteria inside the LBP (43) of the receptor and, consequently, the actual Tm for apo-PPARγ could be lower than the measured value. To avoid these issues, we confirmed our TSA results using nanoDSF as a more accurate technique to quantify the thermal stability of the protein. The nanoDSF results confirmed the TSA structural stabilization profile and maintained the same order for AM-879, P11, and R32 (Table S1 in Supplementary Material), even though the Tm values in the second analysis were more clearly defined. Interestingly, using nanoDSF, the difference between the Tms of apo-PPARγ and PPARγ in the presence of Rosi increased from 1°C to more than 2°C, improving the reliability of the analysis. A possible explanation for this difference is that, in contrast to TSA, which depends on an extrinsic fluorescent dye, nanoDSF measures the intrinsic fluorescence of tryptophan. The use of intrinsic fluorescence avoids, for example, possible undesirable stabilization of the unfolded protein state by the dye used in TSA or by solubilizing agents, such as DMSO (44).

The ANS external probe assay was applied as the second step in our screening pipeline. This technique is a simpler and cheaper solution to identify compounds that successfully compete with the hydrophobic probe in the PPARγ pocket (30). Using this assay, we determined the Kds for the three identified PPARγ ligands (Figure 2). Moreover, based on this assay, we discarded those TSA pre-selected compounds that presented very low affinity for the receptor. In other words, the ANS assay allowed us to reduce the number of compounds in our screening, and indirectly quantified the affinities between PPARγ and the identified ligands. Our results showed that Rosi presented the lowest Kd value. Moreover, R32 presented the lowest affinity for PPARγ among three pre-selected compounds, and P11 and AM-879 showed similar intermediate Kd values.

Another checkpoint of our screening pipeline was the PPARγ cell-based transactivation assay. Interestingly, in this assay, AM-879 and P11 did not present any significant change in PPARγ activation, even at the highest concentration of 0.1 mM (Figure 3), acting as non-agonist ligands. By contrast, R32 activated the receptor at higher concentrations with a submaximal effect compared with activation by the full agonist Rosi; this observation supported the partial agonist activity of this ligand (45). Thus, although AM-879 and P11 have higher affinities for PPARγ compared with R32, they were not able to activate the receptor, which confirmed the relative lack of agonistic properties of these ligands.

Based on this three-step screening, we identified three PPARγ ligands and performed an initial characterization of them, which allowed us to classify them as two non-agonists and one partial agonist. Our objective was to find PPARγ ligands capable of binding to the receptor without promoting any kind of agonism; therefore, we discarded ligand R32 and only further characterized AM-879 and P11. Another piece of evidence that confirmed the efficiency of our screening approach was the identification of AM-879 as a PPARγ ligand. As mentioned before, this same ligand was previously described as a possible PPARγ antagonist, because it reduced the activation of the receptor (29). However, in our assays, we observed a non-agonist action because the basal activation of PPARγ was not changed in the presence of this molecule; for this reason, we further investigated this compound.

Our post-screening characterization of AM-879 and P11, through coregulator recruitment experiments, supported the transactivation assay results. Both ligands have a reduced ability to induce TRAP coactivator recruitment, suggesting that they induce a PPARγ conformation closer to the apo form compared with the Rosi-bound form. In addition, PPARγ in presence of AM-879 presented an affinity for NCOR that was statistically no different to that of the PPARγ apo form (Figure 4B), suggesting that this ligand is not able to displace the NCOR corepressor, maintaining the receptor in a repressed state. In the case of P11, despite its lack of induction of coactivator recruitment, it did not maintain the receptor in the repressed state. For this reason, P11 was discarded. The repressed state is very interesting in terms of finding a promising PPARγ modulator with no obesogenic properties. For this reason, we investigated if the biological effects of AM-879 were related to the effects of classical activation of PPARγ, such as adipogenesis, which had not been previously evaluated.

Evidence from adipocyte differentiation assays showed that AM-879 cannot induce differentiation of 3T3-L1 cells into adipocytes (Figures 5A,B). The maintenance of differentiation levels similar to those of the control, together with transactivation and coregulator recruitment results, strongly suggested the non-agonistic activity of this ligand. In addition, in this cell strain, the previously suggested possible PPARγ antagonist activity of AM-879 (29) was not confirmed since this ligand did not present any significant reduction in adipocyte differentiation compared with untreated control cells, which is a well-described characteristic of antagonists, such as BADGE (46) and GW9662 (47). Interestingly, the inability of AM-879 to induce adipogenesis is actually a desirable characteristic of PPARγ ligands endowed with antidiabetic properties, and may be explained by the relationship between adipogenesis and weight gain produced by TZDs drugs (6, 21, 39, 48, 49).

In the context of adipogenesis, AM-879 did not induce the expression of the adipogenic markers genes, *Cebpa* and *Cd36* (50, 51), in 3T3-L1 cells, which contrasted with the effect of the full PPARγ agonist, Rosi, which, as expected, strongly increased the expression of these genes (9, 45, 52). Cells treated with AM-879 present similar levels of mRNA, in comparison to control cells, for the key regulator of adipogenesis CEBPA, which together with PPARγ, is essential during the early stage of adipocyte differentiation (53), and in the maintenance of this state (54, 55). *Cd36*, another important gene in lipid metabolism that is also a direct target of PPARγ (56), showed slightly reduced mRNA expression after AM-879 treatment. This gene encodes the FA translocase (FAT/CD36) protein, which plays major roles in the transport of long-chain FAs (57). Thus, the data suggested that the decrease in *Cd36* levels produced in response to the ligand might limit the FA influx into adipocytes.

Surprisingly, the expression of the genes encoding adipsin and adiponectin, which are targets of selective modulation by putative antidiabetic ligands, such as L312 (58) and F12016 (52), was also reduced by AM-879 treatment (Figure 5C). It is unclear whether this effect is a consequence of the competition with endogenous PPARγ agonists, such as FAs, leading to possible reduction in PPARγ activation or a consequence of the modulation of other pathways. In the latter case, the CD36 and adiponectin regulation pathways, for example, may be associated, and a decrease in the CD36 level might interfere with the expression of adiponectin (59). In general, these findings showed a tendency of AM-879 to reduce adipogenesis at the genomic level, which agrees with its inability to induce differentiation of 3T3-L1 cells.

As previously demonstrated (6), the insulin-sensitizing effects produced by TZDs are not primarily associated with PPARγ agonism. In fact, the activation of the receptor *per se*, when it induces, for example, adipogenesis, is a putative cause of PPARγ-modulated adverse effects. In this context, phosphorylation of the serine residue S273 in PPARγ LBD was identified as a link between obesity and insulin resistance, and its inhibition by PPARγ ligands, such as TZDs, was directly related to their antidiabetic effects. Interestingly, in addition to the inability of AM-879 to activate PPARγ, this compound also inhibited S273 phosphorylation *in vitro*, suggesting the antidiabetic potential of this ligand; the antidiabetic potential of this ligand may be higher than that of Rosi.

In accordance with these findings, and as demonstrated for other ligands that block phosphorylation (6, 9), our solved crystal structure of PPARγ/AM-879 established that the ligand makes most of these contacts in the β-sheet region of the LBP. In addition, the proximity of this ligand to the amide of S342, in one of its conformations, probably accounts for its capacity to inhibit phosphorylation, because it was reported that the association between the potency of S273 phosphorylation blockage by ligands and the strength of a ligand interaction with S342 amide backbone induces helix 2-helix 2′ loop stabilization (60). In addition, the poor definition of the electron density of the ligand in the crystal structure of the complex PPARγ-LBD/AM-879 may be caused by the limited affinity of this ligand for the protein or may be due to the multiple binding modes that this ligand seems to adopt in the LBP of PPARγ. Interestingly, it has been shown that the sampling of multiple ligand-binding modes could allosterically propagate a conformational disorder to the AF2 surface of the receptor; this allosteric propagation may be sufficient to reduce or even completely block classical agonism (60).

In conclusion, we proposed a new approach to search for PPARγ ligands with partial or non-agonistic properties; this approach comprised a three-step pipeline. We successfully identified three PPARγ hit compounds among a library of 80 molecules. In addition, we were able to characterize the previously described AM-879 ligand experimentally and structurally, as a *bona fide* PPARγ non-agonist with no-adipogenic properties and with the capacity to inhibit CDK5-mediated phosphorylation of PPARγ. Moreover, we demonstrated that AM-879 has a different gene expression regulation profile in adipocytes compared with that of the full agonist Rosi. Together with AM-879, another possible non-agonist, P11, and a partial-agonist, R32, were also identified and may be used as scaffolds for future optimization protocols that may selectively improve their affinity for the PPARγ binding site.

## Author Contributions

Conception and design of the study: AF. Experiments and data collection: HR, NB, AB, TT, JC, FH, and AL. Data Analysis: HR, AL, PO, FH, MB, and NB. Drafting and critical revision of the paper: HR, AL, PO, and MB. Final Revision to be published: HR, AL, and AF.

## Conflict of Interest Statement

The authors declare that the research was conducted in the absence of any commercial or financial relationships that could be construed as a potential conflict of interest.

## Abbreviations

PPARγ, peroxisome proliferator activator receptor γ; LBD, ligand-binding domain; ANS, 8-Anilino-1-naphthalenesulfonic acid; DMSO, dimethyl sulfoxide; FA, fatty acid; Kd, dissociation constant; LBD, ligand-binding domain; Rosi, rosiglitazone; Tm, melting temperature; TSA, thermal shift assay; TZD, thiazolidinediones.
