# Supplementary material for: Screening for PPAR Non-Agonist Ligands Followed by Characterization of a Hit, AM-879, with Additional No-Adipogenic and cdk5-Mediated Phosphorylation Inhibition Properties
Source: Front Endocrinol (Lausanne). 2018 Feb 1;9:11. doi: 10.3389/fendo.2018.00011 (PMC5799700; doi:10.3389/fendo.2018.00011)
Supplement: Supplementary file 1 [file Presentation_1.PDF]

Supplementary Figure 1.

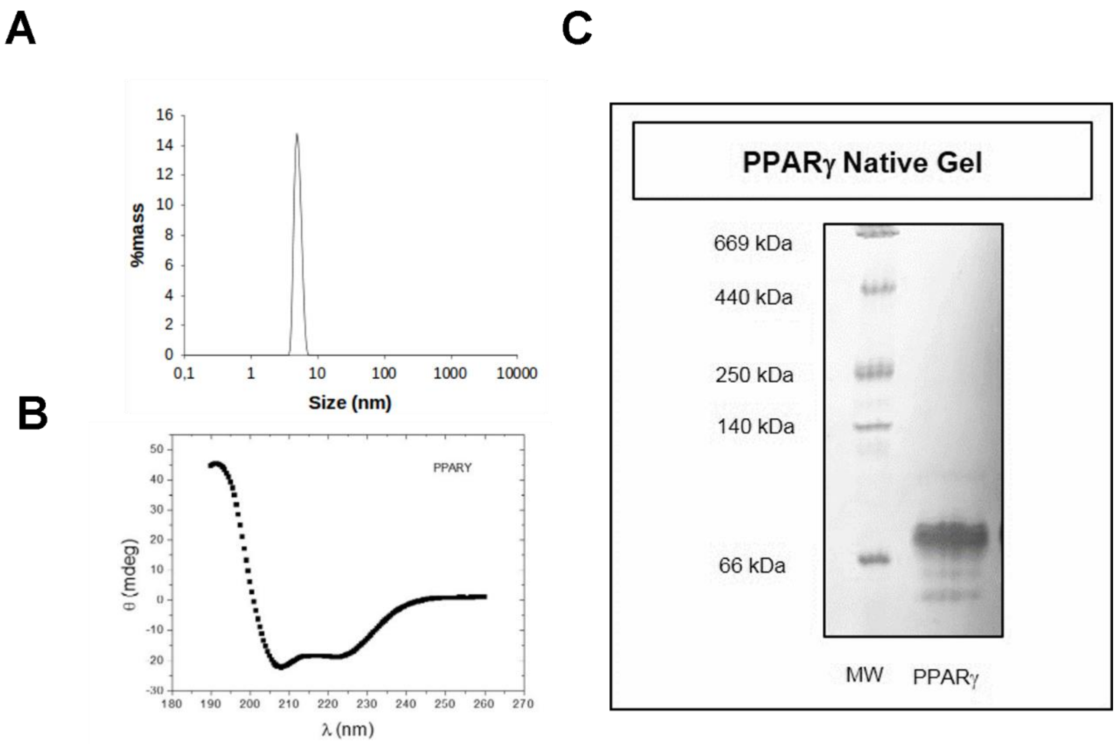

Supplementary Figure 1. **Biophysical characterization and evaluation of PPARγ LBD sample quality.** (A) Distribution of PPARγ LBD size (nm) by percentage of mass obtained in Dynamic Light Scattering (DLS) confirming good quality of PPARγ in monomeric form (Rh = 2.9 nm), with low polydispersion (14,2 %) and with no aggregation. (B) PPARγ spectra recorded using Far-UV Circular Dichroism (CD) from 190 to 260 nm. Two peaks, characteristic of α-helical proteins, are well defined with minimum at 208 and 222 nm. (C) Native gel shows only one band with a good shape which corresponds to PPARγ monomer, indicating also satisfactory packing of the protein tertiary structure.

Supplementary Figure 2.

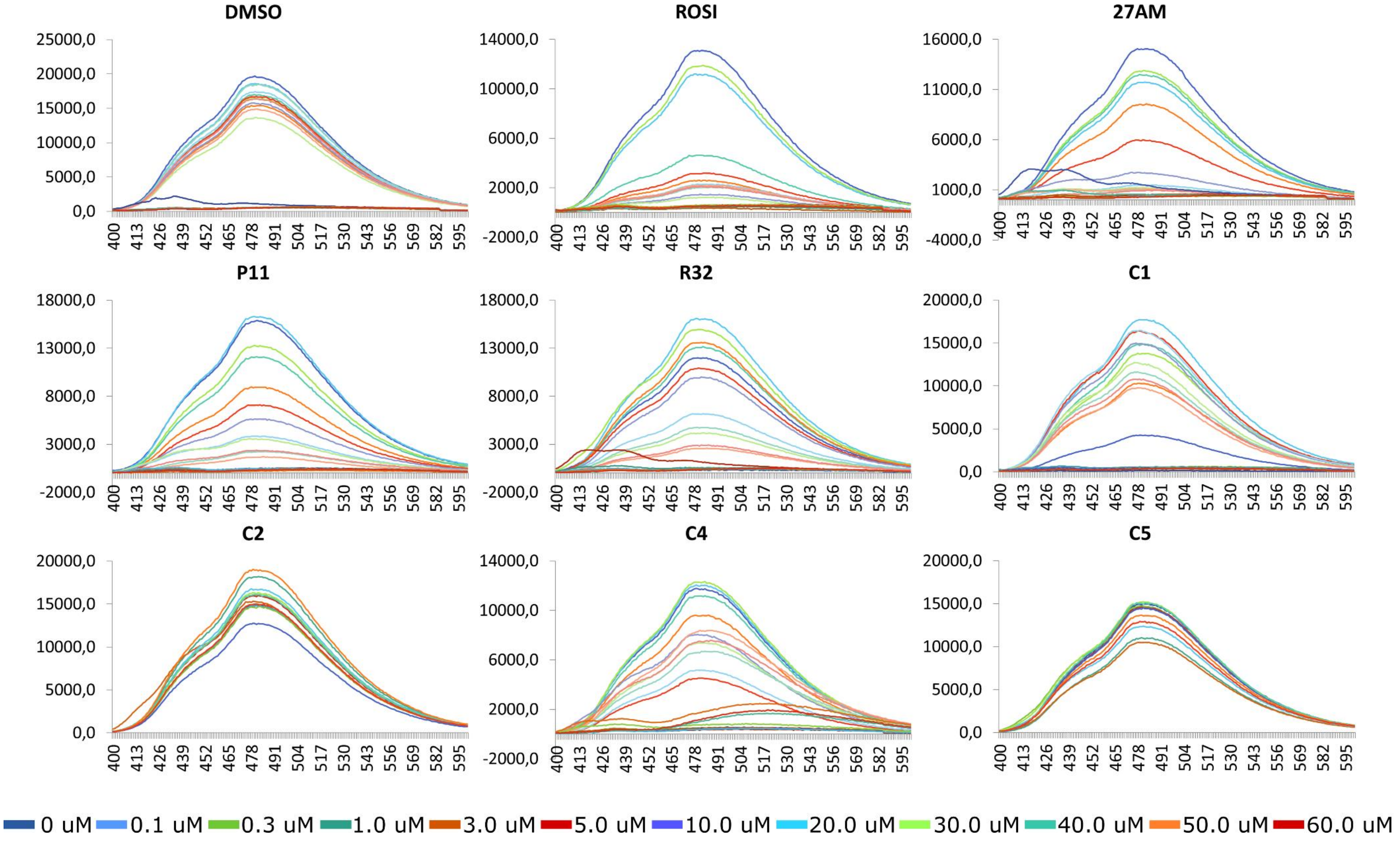

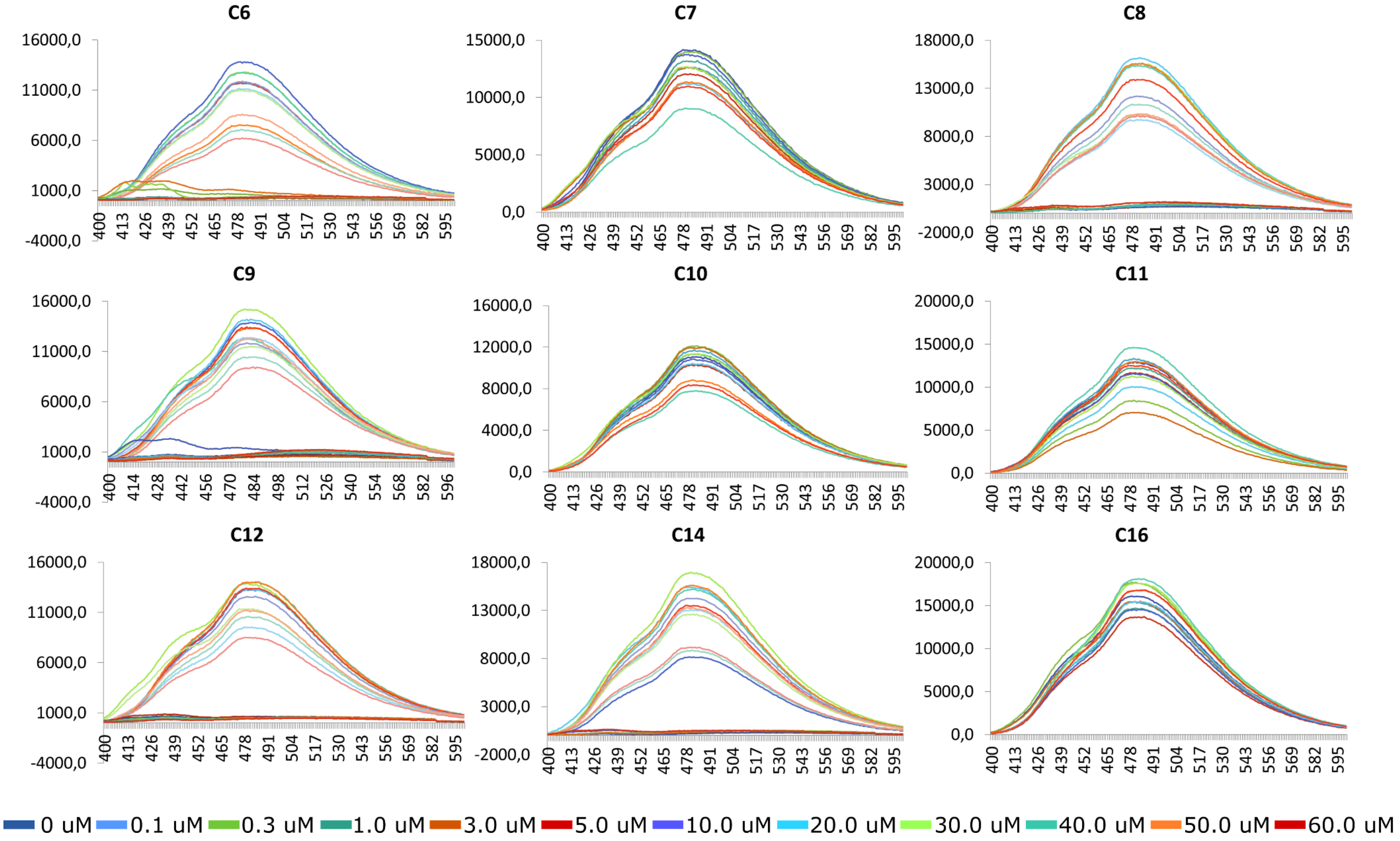

C6
16000,0
11000,0
6000,0
1000,0
-4000,0
400 413 426 439 452 465 478 491 504 517 530 543 556 569 582 595
C7
15000,0
10000,0
5000,0
0,0
400 413 426 439 452 465 478 491 504 517 530 543 556 569 582 595
C8
18000,0
13000,0
8000,0
3000,0
-2000,0
400 413 426 439 452 465 478 491 504 517 530 543 556 569 582 595
C9
16000,0
11000,0
6000,0
1000,0
-4000,0
400 414 428 442 456 470 484 498 512 526 540 554 568 582 596
C10
16000,0
12000,0
8000,0
4000,0
0,0
400 413 426 439 452 465 478 491 504 517 530 543 556 569 582 595
C11
20000,0
15000,0
10000,0
5000,0
0,0
400 413 426 439 452 465 478 491 504 517 530 543 556 569 582 595
C12
16000,0
11000,0
6000,0
1000,0
-4000,0
400 413 426 439 452 465 478 491 504 517 530 543 556 569 582 595
C14
18000,0
13000,0
8000,0
3000,0
-2000,0
400 413 426 439 452 465 478 491 504 517 530 543 556 569 582 595
C16
20000,0
15000,0
10000,0
5000,0
0,0
400 413 426 439 452 465 478 491 504 517 530 543 556 569 582 595
0 uM
0.1 uM
0.3 uM
1.0 uM
3.0 uM
5.0 uM
10.0 uM
20.0 uM
30.0 uM
40.0 uM
50.0 uM
60.0 uM

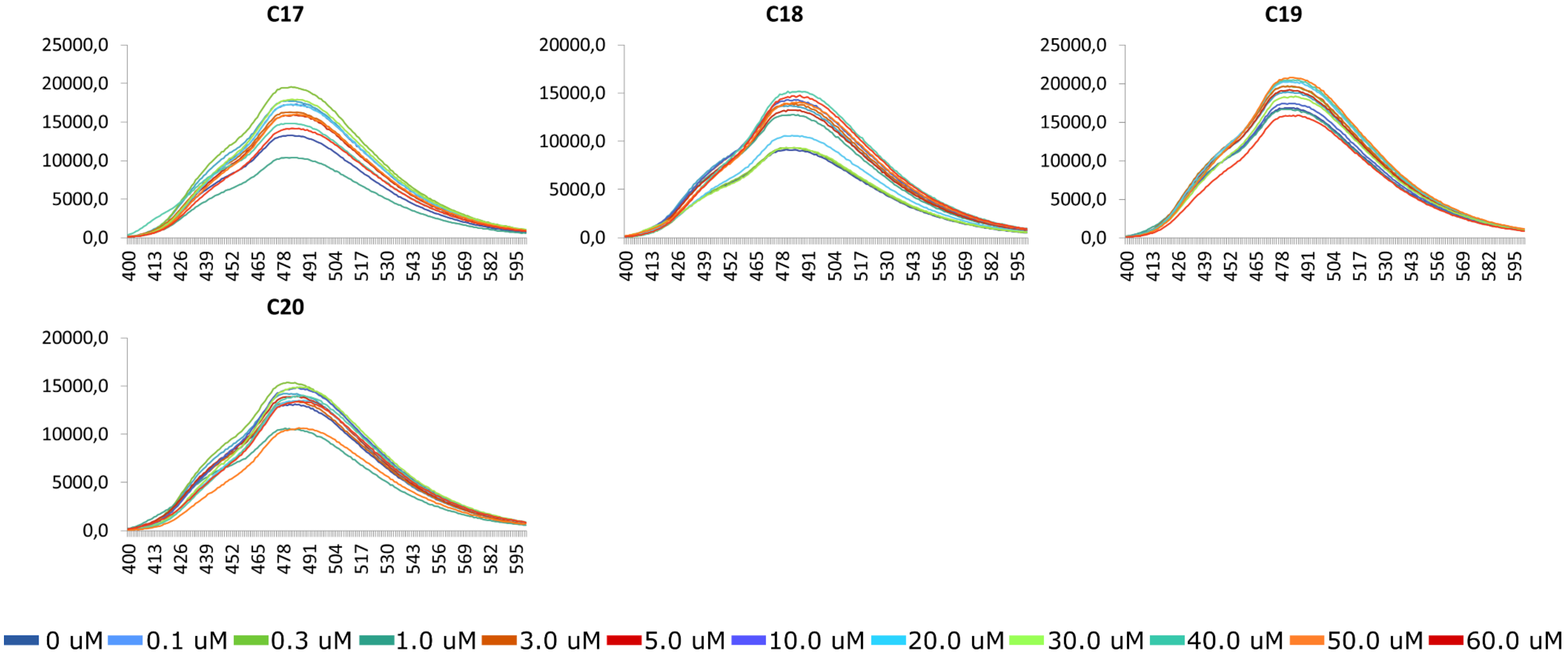

Supplementary Figure 2. ANS fluorescence emission spectra (400 – 600 nm) recorded for PPARγ LBD (1 μM) in the presence of increased concentration of the 20 compounds selected from the TSA screening (0 – 60 μM). Spectra were also recorded in the presence of DMSO or Rosiglitazone, as negative and positive controls, respectively.

Supplementary Table 1.

Ligand	Tm from TSA (˚C)	Tm from nanoDSF (˚C)
DMSO	48.75 ± 0.08	47.3 ± 0.003
Rosiglitazone	49.71 ± 0.05	49.9 ± 0.027
AM-879	49.83 ± 0.01	48.8 ± 0.114
P11	49.4 ± 0.1	48.0 ± 0.172
R32	49.3 ± 0.1	47.9 ± 0.073

Supplementary Table 1. **Melting temperatures of PPARγ LBD** in the presence of DMSO, Rosiglitazone, AM-879, P11 or R32 (3 molar excess), obtained by thermal shift assay (TSA) or employing nanoDSF technique.

Supplementary Figure 3.

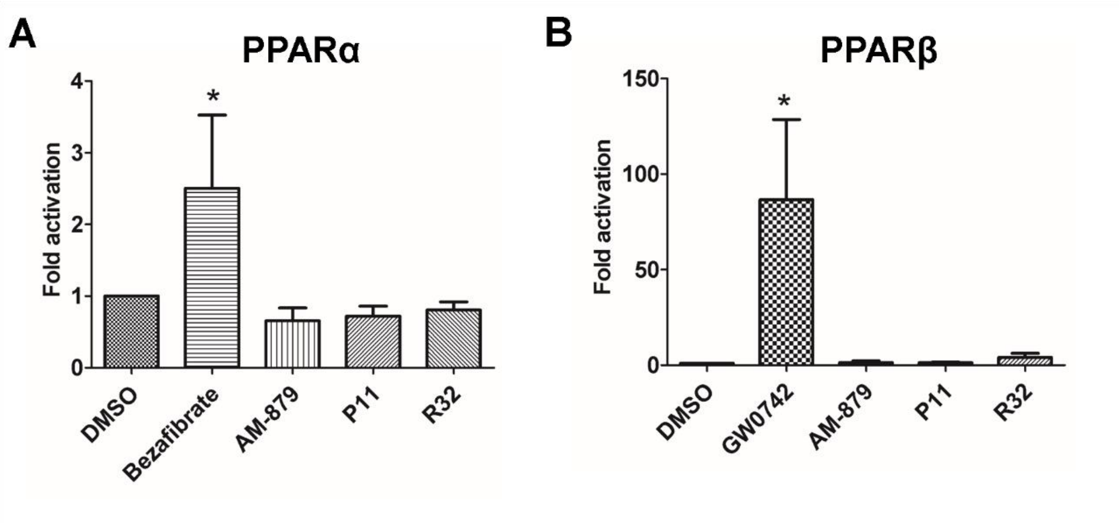

Supplementary Figure 3. PPAR transactivation assay for ligands (1 µM) in HEK 293T cells using a GAL4-PPARα-LBD (A) or GAL4-PPARβ-LBD (B) reporter gene**.** Responses are represented as fold activation normalized against luciferase induction in DMSO. Bezafibrate was used as a full agonist control of PPARα activation and GW0742 as a control of PPARβ activation. Data are the mean ± SEM (n = 5, * $p < 0.05$ *vs* DMSO).

Supplementary Figure 4.

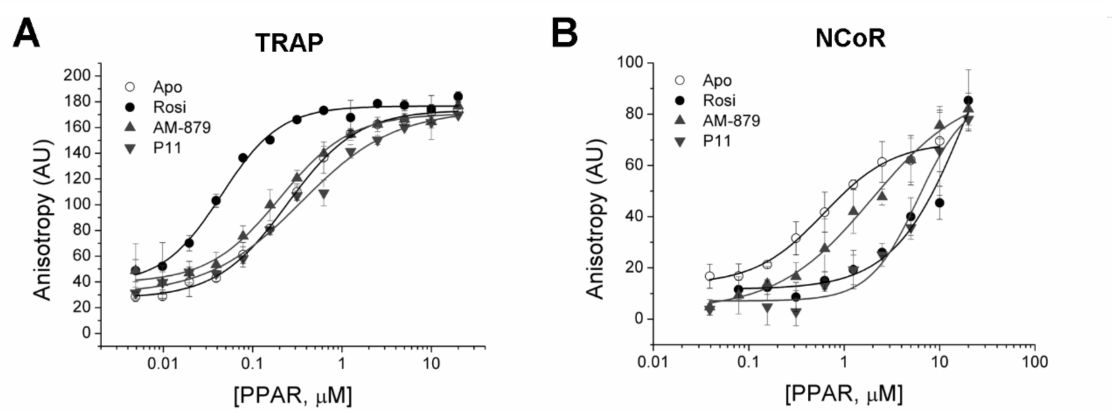

Supplementary Figure 4. Fluorescence anisotropy measurements obtained for the interaction between PPARγ LBD and fluorescein labeled TRAP (ID2) (A) or NCoR (ID2) (B) peptides (20 nM). Curves were measured in the absence (apo, open circles) or in the presence of Rosiglitazone (Rosi, solid black circles), AM-879 (gray up triangles), P11 (down triangles) or R32 (diamond). Data were fitted using the dose-response equation from GraphPad program.

Supplementary Table 2. **Sequences of primers used in RT-PCR experiments.**

Gene	Forward primer	Reverse primer
LPL	GCTGGGCCTAACTTTGAGTATG	TTCTCCTGATGACGCTGATTT
CEBPA	GCGGGAACGCAACATC	GTGCTGGAGTTGACCAGTGAC
PPARg	CACAGAGATGCCATTCTGGC	AGTCAACAGTAGTGAAGGGC
CD36	AAGCTATTGCGACATGATT	GATCCGAACACAGCGTAGAT
Leptin	GAGACCCCTGTGTCGGTTC	CTGCGTGTGTGAAATGTCATTG
Adipsin	CATGCTCGGCCCTACATGG	CACAGAGTCGTCATCCGTCAC
Adiponectin	TGTTCCTCTTAATCCTGCCCA	CCAACCTGCACAAGTTCCCTT

Supplementary Table 3.

PDB CODE	6AN1
**Wavelength (Å)**	1.458620
**Resolution range (Å)**	40.31 - 2.69 (2.786 - 2.69)
**Space group**	C 1 2 1
**Unit cell**	
**a, b, c**	93.3121 61.366 118.45
**α, β, γ**	90 102.65 90
**Total reflections**	120146 (11608)
**Unique reflections**	18370 (1850)
**Multiplicity**	6.5 (6.3)
**Completeness (%)**	99.54 (99.78)
**Mean I/sigma(I)**	20.60 (2.97)
**Wilson B-factor**	66.81
**R-merge**	0.05365 (0.5086)
**R-meas**	0.05833 (0.5553)
**R-pim**	0.02261 (0.2202)
**CC1/2**	0.999 (0.941)
**CC***	1 (0.985)
**Reflections used in refinement**	18319 (1847)
**Reflections used for R-free**	943 (111)
**R-work**	0.2194 (0.2972)
**R-free**	0.2716 (0.3188)
**CC(work)**	0.960 (0.876)
**CC(free)**	0.949 (0.767)
**Number of non-hydrogen atoms**	4141
**Macromolecules**	4071
**Ligands**	58
**Solvent**	12
**Protein residues**	514
**RMS(bonds)**	0.006
**RMS(angles)**	1.26
**Ramachandran favored (%)**	96.23
**Ramachandran allowed (%)**	2.98
**Ramachandran outliers (%)**	0.79
**Rotamer outliers (%)**	2.05
**Clashscore**	12.38
**Average B-factor**	82.16
**Macromolecules**	81.92
**Ligands**	106.37
**Solvent**	49.43
**Number of TLS groups**	16

Supplementary Table 3. Data collection and refinement statistics. Statistics for the highest-resolution shell are shown in parentheses.
